# The impact of a self-selected time restricted eating intervention on eating patterns, sleep, and late-night eating in individuals with obesity

**DOI:** 10.3389/fnut.2022.1007824

**Published:** 2022-10-21

**Authors:** Stacey L. Simon, Jennifer Blankenship, Emily N. C. Manoogian, Satchidananda Panda, Douglas G. Mashek, Lisa S. Chow

**Affiliations:** ^1^Department of Pediatrics, University of Colorado Anschutz Medical Campus, Aurora, CO, United States; ^2^Department of Medicine, University of Colorado Anschutz Medical Campus, Aurora, CO, United States; ^3^Salk Institute for Biological Studies, La Jolla, CA, United States; ^4^Department of Biochemistry, Molecular Biology and Biophysics, University of Minnesota, Minneapolis, MN, United States; ^5^Division of Diabetes, Endocrinology, and Metabolism, University of Minnesota, Minneapolis, MN, United States

**Keywords:** sleep, eating patterns, time restricted eating, intermittent fasting, obesity

## Abstract

**Background:**

Time restricted eating (TRE), limiting eating to a specific daily window, is a novel dietary intervention, but the mechanisms by which TRE results in weight loss remain unclear. The goal of the current study was to examine changes in eating patterns, sleep, and late-night eating, and associations with health outcomes in a secondary analysis of a 12-week self-selected TRE intervention.

**Methods:**

Twenty participants 18–65 years with BMI ≥25 kg/m2 completed the 12-week trial. Participants randomized to TRE (*n* = 11) were instructed to eat during a self-selected 8-h window, while the non-TRE group (*n* = 9) followed their typical eating habits. All participants logged oral intake using the myCircadian Clock mobile application throughout the entire intervention. Anthropometrics, HbA1c, an oral glucose tolerance test, and 2 weeks of actigraphy monitoring were completed at pre-intervention and end-intervention. Independent samples *t*-tests compared differences between groups. Data are presented as mean ± standard deviation.

**Results:**

At preintervention, late night eating was significantly associated with higher fasting glucose (r = 0.59, *p* = 0.006) and higher HbA1c (r = 0.46, *p* = 0.016). The TRE group significantly delayed the timing of the first eating occasion by 2.72 ± 1.48 h relative to wake time (*p* < 0.001) and advanced the timing of the last eating occasion by 1.25 ± 0.8 h relative to bedtime (*p* < 0.001). The non-TRE group, on average, maintained their eating pattern. Sleep measures did not change from pre- to end-intervention, however greater restriction of the eating window was associated with longer sleep duration at end-intervention (β = −0.46 [95% CI −9.2, −0.4], *p* = 0.03). The TRE group significantly reduced the prevalence of late night eating (eating within 2 h of bedtime) by 14 ± 6% (*p* = 0.028) with 63% of participants completely eliminating late night eating at end-intervention.

**Conclusion:**

A self-selected TRE intervention significantly shifted meal timing, reduced late-night eating while prolonging sleep duration.

**Trial registry:**

ClinicalTrials.gov, identifier: 03129581.

## Introduction

Time-restricted eating (TRE), limiting eating to a specific daily window, is emerging as a novel weight management intervention. Several studies have shown that restricting adults with long habitual eating windows (15 or more hours per day) to a food intake window of 8–10 hours per day results in weight loss (1–3). In a randomized control trial, Chow et al. recently reported that a TRE intervention in which individuals self-selected their 8 h eating windows resulted in weight loss (2). Potential mechanisms for successful weight loss with TRE include induced energy deficit, increased energy expenditure, and change in appetite yet very few studies have investigated the impact of TRE intervention on timing of food intake and sleep, which may potentially contribute to TRE-associated weight loss.

Timing of food intake may be an important consideration in TRE interventions. One hypothesis is that TRE allows individuals to synchronize their eating with their circadian rhythms. Evidence suggests that conditions are optimal for food intake in the morning compared to the afternoon/evening, as insulin sensitivity and beta cell responsiveness are higher in the morning than other times of day (4). Similarly, eating during the biological night is associated with metabolic dysregulation (5, 6). Centering the eating window in the mid-day results in weight loss and improvements in insulin sensitivity (1, 7) while shifting the eating window to later in the day may not change or even worsen insulin sensitivity (8–10). In contrast, an isocaloric controlled trial of early TRE (eating window from 8 a.m. to 2 p.m.) resulted in improved insulin sensitivity and beta-cell responsiveness independent of weight change (4). Many TRE studies have assigned the eating window (3, 11, 12). To date, the largest randomized trial of TRE (*n* = 116) assigned late TRE (12-8 pm eating window) and reported no significant difference in weight compared to eating over a 12-h window (12). As self-selection of the eating window may facilitate compliance, it is important to understand how individuals alter their eating during a self-selected TRE intervention and whether the timing of their eating relates to observed weight loss and health outcomes.

Late-night eating, defined as eating dinner within 2 h of bedtime, is associated with an increased risk for cardiometabolic health problems including obesity and metabolic syndrome (13). Large survey studies have found late-night eating habits to be common in several adult populations (14, 15). For example, Yoshida et al. recently reported that in a cohort of 8,153 Japanese adults, 24.6% of participants self-reported eating dinner immediately before bed >3 times per week and 11.9% reported eating snack after dinner (14). Late bedtimes and insufficient sleep duration are associated with obesity in part due to increased evening eating occasions (16). Insufficient sleep is highly prevalent in modern society and independently associated with numerous health consequences including increased weight, excess energy intake, insulin resistance, and risk for type 2 diabetes (17–20). While one study found that TRE improved self-reported sleep quality and daytime alertness (1), other studies found no change in self-reported and objectively-derived sleep duration or timing following TRE (3, 11). However, no prior studies have examined effects of self-selected TRE on the timing of eating relative to bedtime.

We examined changes in timing of eating in relation to sleep following a randomized controlled trial of a 12-week unrestricted eating (non-TRE) vs. restricted eating (TRE: self-selected eating window of 8 h) intervention. We characterized the change in timing of eating and eating patterns from pre- to end-intervention, evaluated whether the TRE intervention reduced late-night eating, and examined associations between timing of eating and health outcomes. We hypothesized that the TRE intervention would reduce late-night eating and that earlier timing of the last eating occasion would be associated with improved metabolic health outcomes.

## Method

This is a secondary analysis of the See Food Study, a randomized controlled trial examining the impact of TRE on body composition and glycemic outcomes (NCT# 03129581). Details of the study have been previously reported in the primary outcomes manuscript (2). The study was approved by the University of Minnesota's Institutional Review Board (IRB) and all participants provided written informed consent before study participation.

### Participants

Participants were aged 18–65 years with BMI ≥ 25 kg/m (2) and an eating window >14 h were recruited from the Twin Cities area through advertisements. They had a stable sleep and work schedule (no shift work, bed and wake times no more than 2 h variance on 6 out of 7 days, and no more than 4 h variance on the 7^th^ day). Participants were excluded if they were pregnant or anticipated becoming pregnant, nursing, or had any significant medical problems such as diabetes or cardiovascular disease based on a screening visit and laboratory evaluation.

### Procedure

After informed consent, participants were randomized to either TRE or non-TRE intervention, stratified by sex and age (±45 years). The TRE group self-selected a daily 8 h eating window, such as 10 am-6 pm or 11 am-7pm, which they were instructed to consistently maintain for the 12 week intervention. During the 8 h eating window, the TRE group could eat *ad libitum*. Outside the eating window, the TRE group could only drink water and take their medications. No other dietary recommendations were given to the TRE group. The non-TRE group was asked to eat *ad libitum* per their typical eating habits. All participants were asked to document all eating occasion (except water or medications) using the mobile application, the myCircadian Clock (mCC: www.mycircadianclock.org) throughout the entire study. Height, weight, body composition, oral glucose tolerance test, and hemoglobin A1c (HbA1c) were obtained at pre-intervention and 12-week end-intervention, and participants wore an ActiGraph Link (ActiGraph, Pensacola, FL) to monitor sleep and activity for 2 weeks just prior to randomization (pre-intervention) and for 2 weeks just prior to study conclusion (week 10–12; end-intervention).

### Measures

#### Meal timing measures

Each participant was instructed to document all oral intake including food, beverages along with an identifying text entry, using the mCC mobile application from the pre-intervention period through Week 12 of the study. The data were time-stamped and adherence to logging (TRE and non-TRE group) and compliance to eating windows (TRE group) were derived from the mCC data. Criteria for adherence have been previously described (2).

Summary metrics of meal timing were determined from the mCC data. As previously described, eating occasions were classified as any food or beverage intake event within 15 min of each other (2). Medication and water were excluded from the analysis. To be included for analysis, all participants were required to have at least 1 day of meal timing data where at least 2 eating occasions (i.e., food or beverages) were logged within a single 24-h period (2).

Timing of eating occasions were expressed as absolute clock time and relative to sleep and wake as defined by actigraphy scoring. The start and end of the eating window were classified into categorical variables using the timing of first and last eating occasion relative to sleep/wake times. These classifications were newly developed for the purpose of the current analyses. The timing of the first eating occasion was used to classify early-starters (first eating occasion ≤ 3.0 h after waking), intermediate-starters (first eating occasion 3.0–5.5 h after waking) and late-starters (first eating occasion ≥ 5.5 h after waking). The timing of the last eating occasion was used to classified early-enders (last eating occasion ≥ 5.5 h before sleep), intermediate-enders (last eating occasion occurring between 3.0–5.5 h before sleep), and late-enders (last eating occasion occurring ≤ 3.0 h before sleep). The categorial classifications of the beginning and end eating windows were combined to create a composite meal timing classification. All potential meal timing classifications are described in the Supplementary Table.

These classifications were also used to approximate different TRE patterns: early-TRE, late-TRE, or intermediate-TRE pattern. Early-TRE eating patterns had eating windows which were concentrated in the early portion of the day (early-starters and early-enders) and was defined as eating primarily in the early portion of the day (first eating occasion ≤ 3.0 h after waking and last eating occasion ≥ 5.5 h before sleep). Late-TRE was defined as eating toward the later portion of the day (first eating occasion occurring ≥ 5.5 h after waking and last eating occasion occurring ≤ 3.0 h before sleep). Intermediate-TRE was defined as delaying the first eating occasion and advancing the last meal of the day (first eating occasion occurring between 3.0–5.5 h after waking and last eating occasion occurring between 3.0–5.5 h before sleep). Eating pattern (early-TRE, late-TRE, intermediate-TRE, or neither) was classified for participants at pre-intervention and end-intervention depending on the eating pattern that was followed for the majority of the time (≥ 50% of valid concurrent mCC app and actigraph data). Finally, we determined the frequency of late-night eating, as defined by eating ≤ 2 h before bedtime, at pre-intervention and end-intervention.

#### Sleep measures

The Actigraph Link (ActiGraph, Pensacola FL), a triaxial accelerometer, was worn on the non-dominant wrist for 2 weeks at pre-intervention and again at end-intervention. Data were collected in 60 second epochs and scored using the Cole-Kripke algorithm. Participants reported their typical weekday and weekend bed and wake times, which were used to facilitate actigraphy scoring. Sleep variables calculated included bedtime, waketime, and total sleep time. The ActiGraph Link has been validated against a previously validated accelerometer for sleep and shown to have a high level of agreement for sleep/wake (96% using the Cole-Kripke algorithm) (21).

#### Anthropometrics and body composition

At pre-intervention and end-intervention, height and weight were measured by study staff. DXA (GE Healthcare Lunar DXA; General Electric Medical Systems, Madison, WI) assessed body composition using standard positioning and imaging protocols.

#### Insulin resistance

Markers of insulin sensitivity were measured at pre-intervention and end-intervention using an oral glucose tolerance test (OGTT) following an 8 h fast. After baseline sampling, 75 g glucose (Trutol 75 Glucose Tolerance Beverage, Thermo Scientific, Waltham, MA) was consumed orally and plasma glucose and serum insulin were sampled every 30 min over 2 h. Homeostatic model assessment of insulin resistance (HOMA-IR) was calculated as [fasting insulin (μU/ml) x fasting glucose (mmol/l)] / 22.5) (22) and Matsuda Index was calculated as √10,000 / [[fasting insulin (μU/ml) x fasting glucose (mmol/l)] x [mean OSTT insulin (μU/ml) x mean OSTT glucose (mmol/l)]] (23) from OGTT results.

### Statistical analysis

An a priori power analysis found that 20 participants (11 in the TRE group and 9 in the non-TRE group) gave 80% power to detect differences in the primary study outcomes [see primary outcomes paper (2)]. Meal timing data with adequate documentation on the mCC app were aligned with sleep data such that intake events during the day were associated with the following night of sleep. If sleep aligned data were not available, the preceding meal timing data were excluded. To be included for analyses, at least 2 days of aligned meal timing data and sleep data were required. All participants provided complete data at pre-intervention (non-TRE: *n* = 9, TRE: *n* = 11). One individual was excluded from the non-TRE at end-intervention because they only provided 1 day of sleep data. In the TRE group, one individual did not have any meals logged during the sleep measurement period. Thus, the end-intervention analysis included 8 individuals in the non-TRE group and 10 individuals in the TRE group.

Demographic characteristics were summarized with descriptive statistics. Independent samples *t*-tests were used to compare pre-intervention demographic, sleep, and meal timing variables between the TRE and non-TRE groups. Linear regression analyses examined associations between intervention group and change in meal timing and sleep, controlling for pre-intervention values. Pearson product moment correlations were also used to examine associations between timing of the last meal and health outcomes at pre-intervention (BMI, HbA1c, fasting glucose, HOMA-IR, and Matsuda Index). SPSS Statistics 26 and R statistical software version 4.0.2 were utilized for analyses. Statistical significance was set at *p* < 0.05.

## Results

### Participant characteristics

Eleven participants completed the TRE intervention (81.8% female, M age = 46.4 ± 12.4 years, M pre-intervention eating window = 15.2 ± 0.8 h), and nine completed the non-TRE intervention (88.9% female, M age = 44.2 ± 12.3 years, M pre-intervention eating window = 15.5 ± 1.1 h). No significant differences were found between groups regarding sex, age, or pre-intervention BMI, insulin sensitivity, or eating window (all *p* > 0.05). No participants were taking any medication for dysglycemia or weight loss treatment during the study. On average, participants had 8 ± 3.0 (mean± SD) valid days (defined as adequate meal documentation on mCC app coupled with adequate concurrent actigraphy data) at pre-intervention and 9 ± 2.5 days at end-intervention. There were no significant between group differences.

### Relationship between late night eating and health outcomes

We evaluated the relationship between late night eating and health outcomes at pre-intervention (Supplementary Figure). Eating close to bedtime was significantly associated with higher fasting glucose (r = 0.59, *p* = 0.006) and higher HbA1c (r = 0.46, *p* = 0.016). There were no other significant associations between health outcomes and the timing of last eating occasions. There were also no significant associations at end-intervention after accounting for pre-intervention values.

### Change in meal timing and eating patterns from pre- to end-intervention

**Figure 1:**
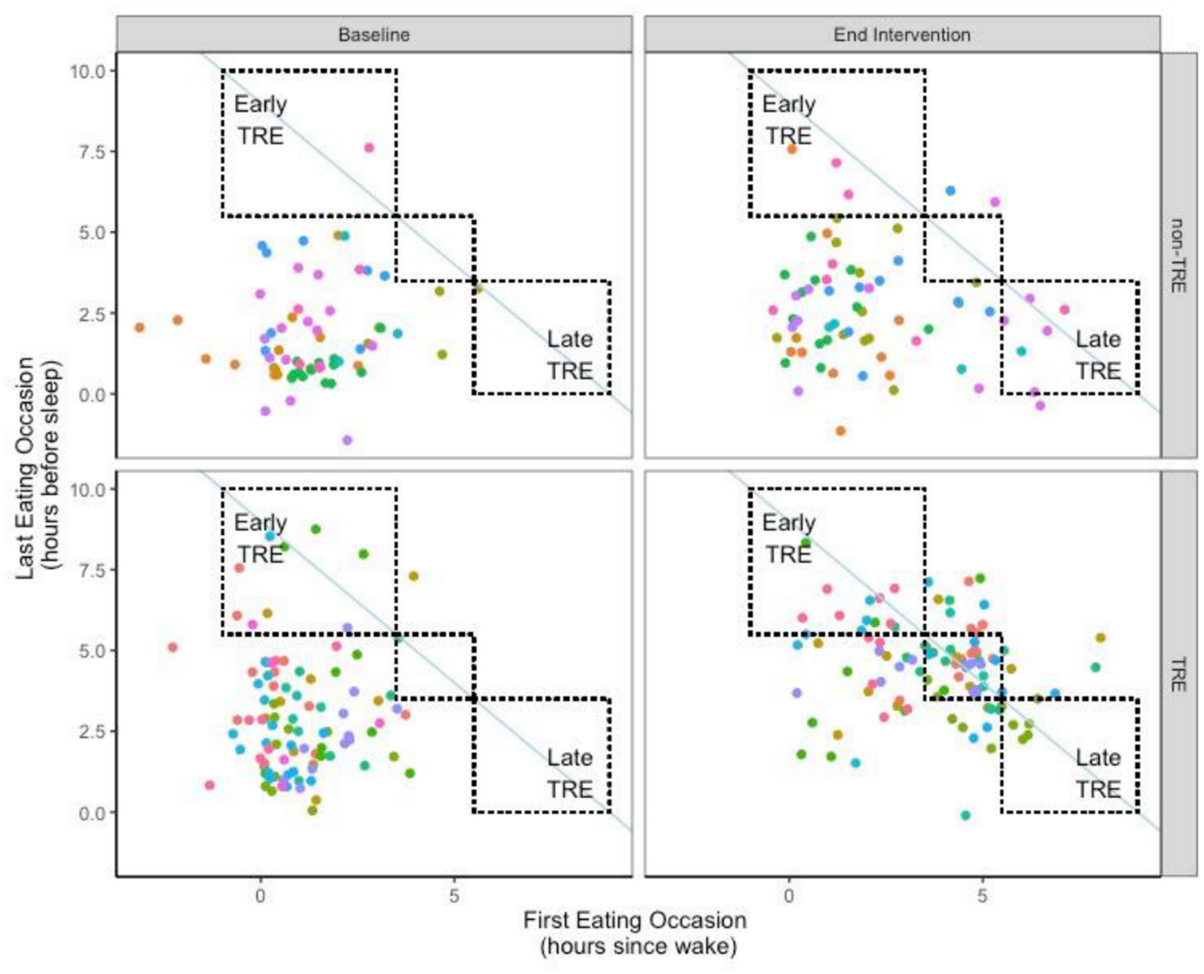
Timing of eating for each day at Pre- and End-Intervention. Individual participants are represented by different colored points. The non-TRE participants are depicted on the top, while the TRE participants are depicted on the bottom; each participant is depicted once at both Baseline **(Left)** and End Intervention **(Right)**. A shift toward the line of identity (blue solid line), would be indicative of a shortened eating window consistent with TRE. **(Upper left)** box represents an early-TRE eating pattern, whereas the lower right box represents a late TRE eating pattern. The middle box represents an intermediate-TRE eating pattern which the timing of the first and last meals were shifted by similar magnitudes.

Between the TRE and non-TRE groups, there were no differences in the timing of eating occasions during the pre-intervention period. At end-intervention, the TRE group significant delayed the time of the first eating occasion of the day, both when expressed by absolute clock time and as hours since wake (Table 1; Figure 1). The TRE group also significantly advanced the last eating occasion of the day (mean change:−1.5 ± 0.8 h). In contrast, the timing of the first and last eating occasions in the non-TRE group remained similar between pre- to end-intervention.

**Table 1:** Meal timing of first and last eating occasions.

	**TRE (*****n*** = **11)**			**Non-TRE (*****n*** = **9)**			
	**Pre-intervention** **(*n* = 11)**	**End-intervention** **(*n* = 10)**	***P* (time)**	**Pre-intervention** **(*n* = 9)**	**End-intervention** **(*n* = 8)**	***P* (time)**	*P* (group*time)
First (absolute clock time)	07.55 ± 01.22	10.47 ± 01.17	<0.001	08.09 ± 01.09	09.02 ± 02.23	0.20	0.016
First (hours since wake)	1.08 ± 0.78	3.82 ± 1.19	<0.001	1.48 ± 1.50	2.56 ± 2.24	0.08	0.051
Last (absolute clock time)	20.10 ± 01.08	18.31 ± 1.04	<0.001	21.00 ± 1.43	20.25 ± 1.25	0.16	0.058
Last (hours before sleep)	−3.25 ± 0.76	−4.52 ± 0.72	<0.001	−2.93 ± 1.65	−2.93 ± 1.55	0.99	0.015
Midpoint eating (clock time)	14.02 ± 00.49	14.39 ± 01.04	0.38	14.38 ± 00.59	14.28 ± 01.39	0.80	0.34

Meal timing is expressed as absolute clock time and relative to sleep and wake timing. Data are presented as mean ± standard deviation.

Eating pattern was categorized using the timing of the first and last eating occasion of the day (Table 2). At pre-intervention, the majority of participants in both groups (*n* = 17, 85%) started eating early in the day and ended eating late in the day (i.e., Early-Late eating pattern). At end intervention, most of the participants in the TRE group (*n* = 6, 60%) did not follow a consistent eating pattern. One individual in the TRE group shifted to a consistent Early-TRE eating pattern such that they began eating early in the day and finished eating early in the day on 55% of days. There were 3 individuals who followed a consistent Intermediate-TRE eating pattern where the first eating occasion was moderately delayed, and the last eating occasion was moderately advanced, on 54 ± 7% of days. Finally, no participants in the TRE group elected to follow a Late-TRE pattern. While most individuals (*n* = 5, 56%) in the non-TRE group maintained the Early-Late eating pattern at end intervention, there were 2 individuals who shifted to an inconsistent eating pattern in non-TRE. Finally, there were 2 individuals in the non-TRE group who adopted a Late-TRE eating pattern where they delayed both their first and last eating occasions.

**Table 2:** Meal timing patterns.

	**TRE (*****n*** = **11)**		**Non-TRE (*****n*** = **9)**	
**Meal Timing Pattern**	**Pre (*n* = 11)**	**End (*n* = 10)**	**Pre (*n* = 9)**	**End (*n* = 9)**
Early-Early (Early TRE)	0	1	0	0
Early-Intermediate	1	0	1	0
Early-Late (Unrestricted)	8	0	7	5
Intermediate-Intermediate (Intermediate-TRE)	0	3	0	0
Late-Late (Late-TRE)	0	0	0	2
Inconsistent	2	6	1	2

Eating patterns participants followed for the majority (≥ 50%) of the measurement period. Early-Early: first eating occasion ≤3.0 hours after wake and last eating occasion ≥5.5 h before sleep. Early-Intermediate: first eating occasion ≤3.0 h after wake and last eating occasion between 3.0–5.5 h before sleep. Early-Late: first eating occasion ≤3.0 h after wake and last eating occasion ≤3.0 h before sleep. Intermediate-Intermediate: first eating occasion between 3.0–5.5 h after wake and last eating occasion between 3.0–5.5 h before sleep. Late-Late: first eating occasion ≥5.5 h after wake and last eating occasion ≤3.0 h before sleep. Inconsistent: Participants who did not follow one eating pattern for ≥50% of the measurement period were classified as inconsistent.

### Change in late-night eating from pre- to end-intervention

We investigated change in late-night eating, or the timing of the last eating occasion relative to bedtime (expressed as hours before bed) from pre- to end-intervention. At pre-intervention, all participants had at least one instance of eating within 2 h of bedtime. The non-TRE group ate within 2 h of bedtime 57 ± 19% of the time, whereas the TRE group had significantly fewer instances of late-night eating at pre-intervention (TRE: 36 ± 13% of the time, group difference at pre-intervention *p* = 0.014). At end-intervention, there was a significant reduction in late-night eating in the TRE group (14 ± 6%, *p* = 0.028) and all but 4 participants in the TRE group completely eliminated eating within 2 h of bedtime. The non-TRE group did not significantly change late-night eating habits compared to pre-intervention, with 9 individuals performing late-night eating 43 ± 27% of the time (*p* = 0.068). Change in late-night eating was not significantly correlated with sleep duration (*p* > 0.05).

### Change in sleep variables from pre- to end-intervention

Pre-intervention values and change in actigraphy-estimated sleep variables are reported by group in Table 3. Compared to the recommended 7–9 h (24), participants in both groups on average obtained insufficient sleep on weekdays and weekends at pre-intervention and end-intervention. Average sleep duration at pre-intervention for the entire sample was 6.32 ± 0.55 h, and 6.29 ± 0.49 h at end-intervention. No significant differences in weekday or weekend sleep variables were found between the TRE and Non-TRE groups at either pre-intervention or end-intervention. Changes in all actigraphy variables from pre- to end-intervention were not significantly associated with group. Analyses revealed a significant association between sleep duration and change in eating window, controlling for pre-intervention eating window. Specifically, greater restriction of the eating window was associated with longer end-intervention sleep duration, after accounting for pre-intervention values (β = −0.46 [95% CI −9.2, −0.4], *p* = 0.03).

**Table 3:** Pre-intervention and change from pre- to end-intervention for actigraphy-estimated sleep by group.

	**TRE (*****N*** = **11)**				**Non-TRE (*****N*** = **9)**			
	**Pre-Intervention**		**End-Intervention** **(Change from pre-)**		**Pre-Intervention**		**End-Intervention** **(Change from pre-)**	
	**Weekday**	**Weekend**	**Weekday**	**Weekend**	**Weekday**	**Weekend**	**Weekday**	**Weekend**
Total Sleep Time (h:min)	6.21 ± 0.35	6.06 ± 1.03	0.04 ± 0.35	0.23 ± 0.54	6.16 ± 0.38	6.28 ± 0.56	−0.16 ± 0.24	0.06 ± 1.01
Bedtime (clock time)	23.01 (21.34, 24.02)	23.19 (22.13, 24.41)	−7.4 ± 18.8 min	−8.6 ± 35.1 min	22.38 (21.08, 23.58)	23.20 (22.02, 02.31)	7.0 ± 33.2 min	−50.5 ± 62.3 min
Waketime (clock time)	06.39 (05.37, 08.11)	07.03 (05.41, 08.32)	6.4 ± 33.6 min	12.9 ± 39.1 min	06.29 (05.22, 08.01)	07.29 (06.20, 10.38)	−3.0 ± 30.6 min	−42.3 ± 69.4 min

Data are presented as mean ± SD or mean (range). No significant differences were found between groups for pre-intervention sleep variables (all p > 0.05). Between-groups change from pre- to end-intervention was non-significant for all sleep variables (all p > 0.05).

## Discussion

In this examination of participants following a randomized-controlled trial of a self-selected TRE intervention, we found that the TRE group significantly delayed the first eating occasion of the day and advanced the last eating occasion of the day, with most participants following an Early or Intermediate TRE eating pattern in the TRE group. In contrast, most participants in the non-TRE group maintained an Early-Late eating pattern such that their food consumption occurred across the entire day. Notably, only 4 participants in the TRE condition were consistent across days in their eating pattern, while most participants had a combination of both Early and Intermediate eating.

Findings from the current study suggest that TRE intervention does not worsen sleep, as we found no change in objectively measured sleep duration or timing from pre- to end-intervention for either group. Moreover, greater restriction of the eating window was associated with longer sleep duration. However, study participants on average obtained less than the recommended 7–9 h of sleep per night, suggesting insufficient sleep. As insufficient sleep is independently associated with obesity, insulin resistance, type 2 diabetes, and metabolic syndrome (25–27), a focus on TRE's effects on sleep may be warranted. Future research incorporating behavioral strategies to enhance sleep during TRE intervention could be considered.

Late-night eating was significantly reduced in the TRE group, with participants completely ceasing food consumption within 2h of bedtime at end-intervention. This was in contrast to the non-TRE group which did not change their late-night eating habits. Prior studies have shown that late-night eating is associated with obesity, dyslipidemia, hyperglycemia, and metabolic syndrome (28), consistent with our findings that late-night eating was associated with higher fasting glucose and HbA1c. Sutton et al. investigated the effects of an early TRE intervention (6-h eating window which the end time was set to 15:00) on weight and metabolic outcomes. Despite no change in weight, they found improvements in insulin sensitivity, beta cell responsiveness, and blood pressure (4). In contrast, Weiss recently showed that in a TRE intervention which restricted the eating window to 12:00–20:00, there were no changes in glycemic outcomes (12). Data from the present study are consistent with previous reports in the literature. Together, these findings suggest that reduced late-night eating may be one mechanism by which TRE might improve metabolic measures. Further studies on the timing of TRE are warranted to understand which components of TRE interventions drive the reported weight and metabolic related changes.

The field of TRE suggests restricting eating to a certain time of day affects daily activity-rest rhythms and the intrinsic circadian clock (29). Specifically, TRE may alter the circadian misalignment that commonly occurs in today's society due in part to activity and eating through the day and night facilitated by artificial light and the prevalence of personal electronic devices (30). Because participants in the current study self-selected their eating window, it is possible that they chose their TRE schedule based on their convenience, regardless of their natural circadian rhythm. A longitudinal study of the British Birth Cohort showed that more irregular eating patterns were associated with future risk for metabolic syndrome and obesity (31). Others have demonstrated that consistency in the timing of other lifestyle behaviors, like exercise, is related to improved weight loss (32), thus considering the variability of behavior timing may be an important consideration for future studies. Notably, 6 participants were classified as having an Inconsistent meal timing pattern at end-intervention. Further study considering the impact of meal timing variability in the context of TRE and impact on health outcomes is suggested. Additionally, future research assessing chronotype (morning vs. evening preference) and circadian rhythms directly *via* melatonin in conjunction with both self-selected and assigned TRE schedules may help to better elucidate how TRE interacts with circadian rhythms to effect weight loss.

Strengths of the current study include objective measurement of sleep variables, real-time assessment of dietary timing, and a randomized, age- and sex-matched non-TRE control group. Having the control group provided critical comparison, as several participants still shifted their eating patterns despite being instructed to maintain their usual eating habits. Limiting factors include the small sample size, which limited our power and reduced our ability to control for potential covariates in analyses, though the current sample is similar to many of the existing TRE studies (3, 11). As food intake was captured only by images and text description in the mCC app, we could not quantify energy intake, another acknowledged limitation. Thus, interpretation of results should be considered in the context of this preliminary pilot study. A majority of participants were female, which may limit generalizability of findings to males. Part of the inclusion criteria for the current study required participants to have a relatively stable sleep schedule; thus, our findings may not be representative of those with more variable schedules. It is worth noting that 2 individuals in the non-TRE group shifted their eating schedule such that they followed a late TRE eating pattern, suggesting that just by tracking energy intake and/or being involved in a study assessing the impact of TRE may influence eating behaviors. These data emphasize the importance of assessing adherence to intervention and control group requirements in behavioral interventions particularly in those interventions which are popular in the mainstream media.

## Conclusion

In summary, our data show that during self-selected TRE (goal of 8 h window) most participants followed an Early and/or Intermediate eating pattern and significantly reduced their late-night eating. The TRE intervention did not negatively impact sleep, and those with greater restriction of their eating window also had longer sleep duration. We recommend applying the meal timing classification methodology to future trials of TRE in larger sample sizes. Future research examining eating variability, directly intervening on sleep habits, and comparing Early vs. Late TRE, as well as Intermediate TRE as a novel framework in larger samples may help elucidate mechanisms responsible for the effect of TRE on weight loss in individuals with obesity.

## Data availability statement

The raw data supporting the conclusions of this article will be made available by the authors, without undue reservation.

## Ethics statement

The studies involving human participants were reviewed and approved by University of Minnesota IRB. The patients/participants provided their written informed consent to participate in this study.

## Author contributions

LC, SS, and JB: conceptualization. LC, DM, SP, and EM: methodology. SS and JB: formal analysis and writing–original draft preparation. LC: investigation and funding acquisition. SS, JB, EM, SP, DM, and LC: writing–review and editing. All authors have read and agreed to the published version of the manuscript.

## Funding

This work was supported by the Healthy Foods Healthy Lives program (17SFR-2YR50LC to LC) and the National Institutes of Health (NIH National Center for Advancing Translational Sciences, UL1TR002494; NIH NIDDK, 1K23DK117021 to SS). The funders had no role in the design of the study; in the collection, analyses, or interpretation of data; in the writing of the manuscript, or in the decision to publish the results.

## Conflict of interest

Author SP has authored the book The Circadian Code, for which he receives author royalties and in which he specifically recommends time restricted eating. The remaining authors declare that the research was conducted in the absence of any commercial or financial relationships that could be construed as a potential conflict of interest.

## Publisher's note

All claims expressed in this article are solely those of the authors and do not necessarily represent those of their affiliated organizations, or those of the publisher, the editors and the reviewers. Any product that may be evaluated in this article, or claim that may be made by its manufacturer, is not guaranteed or endorsed by the publisher.
