# Supplementary material for: The impact of a self-selected time restricted eating intervention on eating patterns, sleep, and late-night eating in individuals with obesity
Source: Front Nutr. 2022 Oct 21;9:1007824. doi: 10.3389/fnut.2022.1007824 (PMC9634110; doi:10.3389/fnut.2022.1007824)
Supplement: Supplementary file 1 [file Table_1.PDF]

Supplemental Table: Meal Timing Classifications

		**First Eating Occasion**		
**Last Eating Occasion**		**Early-starter** (≤ 3.0 h after wake)	**Intermediate-starter** (3.0-5.5 hours after wake)	**Late-starter** (≥5.5 hours after wake)
	**Early-ender** (≥5.5 hours before sleep)	Early-TRE	Intermediate-Early	Late-Early
	**Intermediate-ender** (3.0-5.5 hours before sleep)	Early-Intermediate	Intermediate-TRE	Late-Intermediate
	**Late-ender** (≤3.0 hours before sleep)	Unrestricted	Intermediate-Late	Late-TRE

Note. Classifications of all potential meal timing patterns derived from the timing of the first and last eating occasion relative to wake and sleep timing. Eating start time (columns) indicates the time at which eating began relative to wake time. Early-starter: ≤3.0 hours after wake, Intermediate-starter: 3.0-5.5 hours after wake, Late-starter: ≥5.5 hours after wake. Eating end (rows) indicates the time at which eating ended before sleep time. Early-ender: ≥5.5 hours before sleep, Intermediate-ender: 3.0-5.5 hours before sleep, Late-ender: ≤3.0 hours before sleep. Grey boxes indicate meal timing patterns that were not observed in the sample of the current study.
