# Supplementary figures and images for: The impact of a self-selected time restricted eating intervention on eating patterns, sleep, and late-night eating in individuals with obesity
Source: Front Nutr. 2022 Oct 21;9:1007824. doi: 10.3389/fnut.2022.1007824 (PMC9634110; doi:10.3389/fnut.2022.1007824)

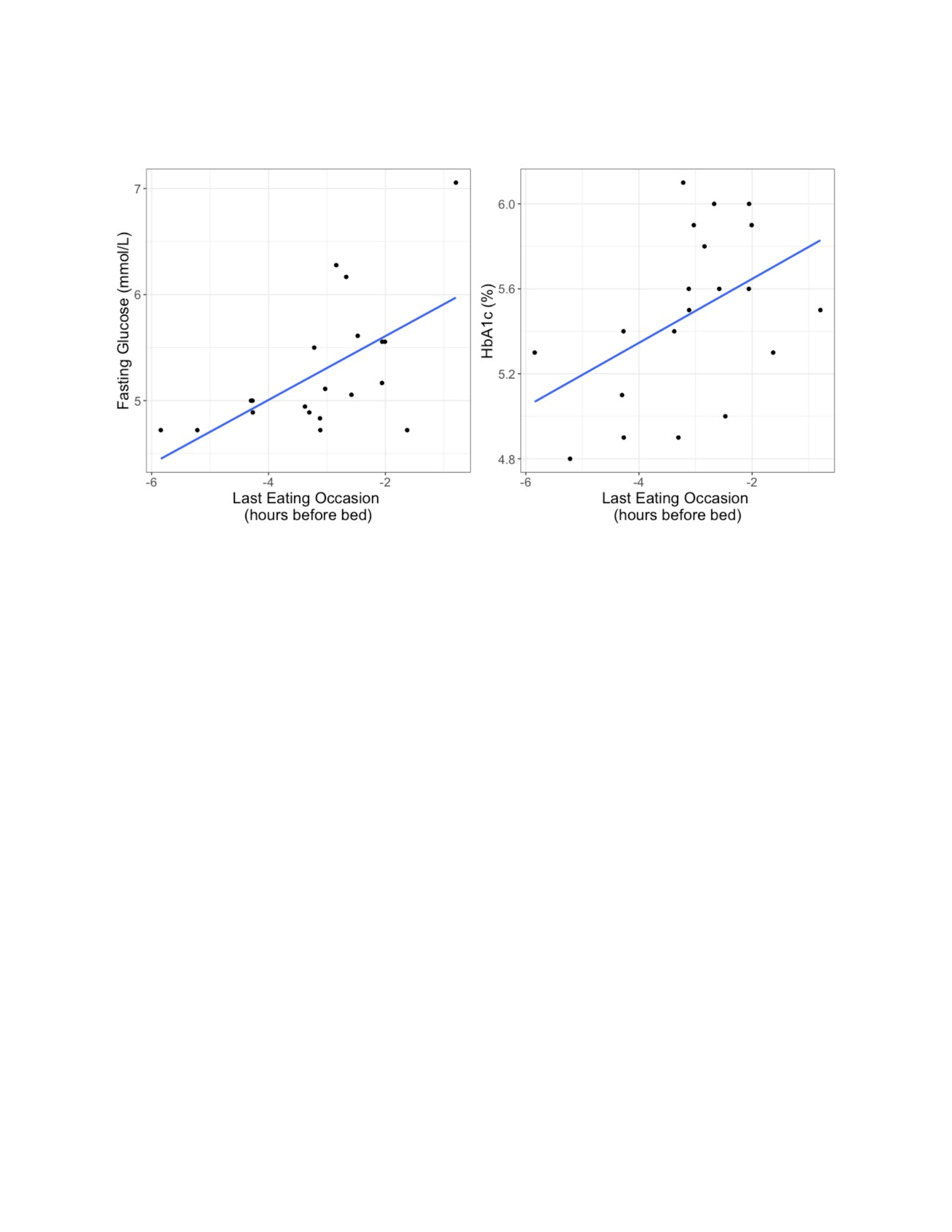

Supplement: Supplementary file 2 [file Image_1.tif]
